# Architecture for Knowledge-Based and Federated Search of Online Clinical Evidence

**DOI:** 10.2196/jmir.7.5.e52

**Published:** 2005-10-24

**Authors:** Enrico Coiera, Martin Walther, Ken Nguyen, Nigel H Lovell

**Affiliations:** ^1^Centre for Health InformaticsUniversity of New South WalesSydneyAustralia; ^2^Graduate School of Biomedical EngineeringUniversity of New South WalesSydneyAustralia

**Keywords:** Evidence-based medicine, clinical decision support systems, information retrieval, meta-search filters

## Abstract

**Background:**

It is increasingly difficult for clinicians to keep up-to-date with the rapidly growing biomedical literature. Online evidence retrieval methods are now seen as a core tool to support evidence-based health practice. However, standard search engine technology is not designed to manage the many different types of evidence sources that are available or to handle the very different information needs of various clinical groups, who often work in widely different settings.

**Objectives:**

The objectives of this paper are (1) to describe the design considerations and system architecture of a wrapper-mediator approach to federate search system design, including the use of knowledge-based, meta-search filters, and (2) to analyze the implications of system design choices on performance measurements.

**Methods:**

A trial was performed to evaluate the technical performance of a federated evidence retrieval system, which provided access to eight distinct online resources, including e-journals, PubMed, and electronic guidelines. The Quick Clinical system architecture utilized a universal query language to reformulate queries internally and utilized meta-search filters to optimize search strategies across resources. We recruited 227 family physicians from across Australia who used the system to retrieve evidence in a routine clinical setting over a 4-week period. The total search time for a query was recorded, along with the duration of individual queries sent to different online resources.

**Results:**

Clinicians performed 1662 searches over the trial. The average search duration was 4.9 ± 3.2 s (N = 1662 searches). Mean search duration to the individual sources was between 0.05 s and 4.55 s. Average system time (ie, system overhead) was 0.12 s.

**Conclusions:**

The relatively small system overhead compared to the average time it takes to perform a search for an individual source shows that the system achieves a good trade-off between performance and reliability. Furthermore, despite the additional effort required to incorporate the capabilities of each individual source (to improve the quality of search results), system maintenance requires only a small additional overhead.

## Introduction

Clinicians need to keep up-to-date with the biomedical literature in order to practice according to the best available evidence. However, this has become increasingly difficult as the amount of medical literature a clinician needs to consider grows exponentially [1,2]. As a result, the effort required to find a specific piece of evidence increases year after year [3]. Clinicians typically work under time pressure, which compounds the problem. The need to develop robust methods and tools to support evidence access is now widely recognized. Online evidence retrieval methods are increasingly seen as a core tool in support of evidence-based health care [4]. In the traditional model of online evidence services, clinicians have access to a number of online information sources, such as journals, databases, and Medline, each with its own idiosyncrasies and search interfaces. This means users need to know which resources are most suitable for their current question and how the search query must be formulated for a given resource. Interoperability standards for the efficient dissemination of content are being developed (eg, the Open Archive Initiative [5]), but until the majority of content adheres to such standards, there is still a need to search through heterogenous data sources.

The meta-search engine approach [6,7] addresses many of the limitations of these models by providing a mechanism to search all the available resources at one time and by translating user queries into the respective query languages of each resource. This typically uses a least-common-denominator approach, directly passing on user keywords to different information sources without regard for the specific capabilities or limitations of these resources. For example, a meta-search engine often disregards the rich query language available with some resources in order to simplify the overall meta-search process. Consequently, while the user expects the meta-search to return an integrated set of search results, the reality is that some resources would have been able to perform much better had they been queried individually; the user is unaware of the variations in search quality across the different resources that have been queried for them. Variants of the standard meta-search engine approach have been shown [8] to provide search capabilities beyond the least common denominator but still require users to select the resources they wish to search. One solution to this problem is to “federate” the different resources so that they more genuinely behave as one uniform data source. A federated search system may perform a *syntactic reformulation* of a user query, translating it into queries that have been optimized for the native query language of individual evidence sources. *Semantic reformulation* is also possible [9]. For example, user keywords may be translated into equivalent keywords or phrases using a terminological system.

However, a federated search can still produce an excessive number of candidate documents, or hits, many of them failing reasonable tests of relevance. One way to improve the chance of retrieving clinically relevant information is to pre-program a search system with specialist bibliographic knowledge using *search filters*. Search filters capture expert strategies for searching that are known to improve the precision of searches. For example, Medline offers a small set of “clinical queries,” which are pre-defined and validated search filters optimized to retrieve documents that are most likely to be clinically relevant, emphasizing disease etiology, diagnosis, therapy, or prognosis [10,11]. Such search filters are necessarily highly customized to the capabilities of individual information sources and their native search engines. For a federated search system to consistently use search filters, it would need to develop a generalized approach to search filters, or *meta-search filters*. Quick Clinical (QC) [4] is a federated evidence retrieval system designed to meet the specific needs of clinicians. Its design incorporates the novel use of meta-search filters to optimize search strategies, and it is based upon a wrapper-mediator architecture built around a universal query language. This paper describes the system architecture of QC and the technical challenges to the design of online evidence retrieval systems, and it reports on the technical performance of the system from a clinical trial with primary care physicians.

## Methods

### The Quick Clinical System

#### User Interface

**Figure 1:**
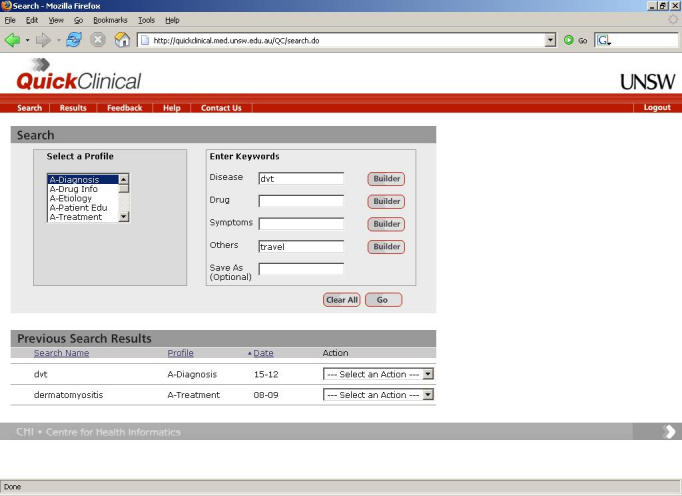
The QC search query user interface

In the QC user model, a user is presented with a single query interface, which connects to an arbitrarily large number of federated knowledge sources and incorporates query specific meta-search filters called “profiles.” QC guides users to first consider the purpose of their search through selection of a profile, and it then asks them to provide specific keywords related to that search task. As a consequence, users are guided through a process that structures their query for them and improves the chances that they will ask a well-formed query and receive an appropriate answer. Figure 1 depicts the QC search interface. On the left hand is a list of search filters that describe typical search tasks and that are customized to the specific information needs of different user groups. Figure 1 shows filters specifically designed for use by primary care physicians.

**Figure 2:**
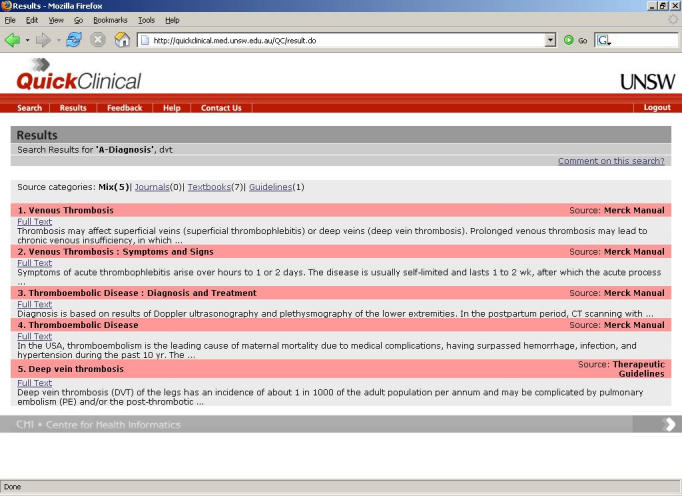
Screenshot of a QC results page

In QC, individual profiles are able to define different keyword types, such as “disease,” which describe the keyword classes typically associated with that profile. Thus, on the right of the interface are four fields where users can provide keywords describing the specific attributes of their search. Selection of a different profile may thus alter the keyword types requested from the user for a given search. QC then translates and submits search queries to the sources specified in the chosen profile, collects and processes the results, and presents them to the user as a list of documents (Figure 2). The title of a document is followed by the link and a short abstract of the content. A user can drill-down into a specific group of results by source type (eg, journal articles or guidelines).

#### Quick Clinical System Architecture

##### Overview

**Figure 3:**
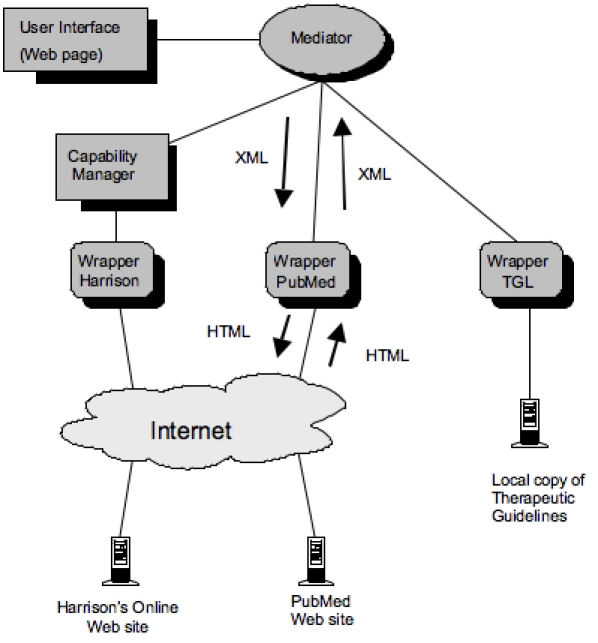
Architecture overview of Quick Clinical

Most information sources such as websites, online texts, and databases have their own proprietary search interface, including query language and format for the display of results. Therefore, a federated meta-search engine that wishes to query a number of different information sources needs to first represent a user query using some internal query language [13] and then translate that internal query into the specific query languages of the relevant data sources. A well-documented [12] approach to this problem is to use a “wrapper” (Figure 3), which acts as an adapter between the proprietary language of individual information sources and the internal language used within a meta-search system. In QC, the internal query language is called the unified query language (UQL). Each information source known to QC has its own wrapper that translates queries from UQL into the native language of the source. As a result, internal components of QC only need to know UQL and not the individual query languages of the data sources. System maintenance is also simplified since the introduction of a new data source to the system only requires one new wrapper component to be generated. Once the results of a search are returned by an information source, the information must again be translated into a standard output format for presentation to the user, which, in QC, is called the unified response language (UReL). UReL also allows other components in the system to modify the presentation of search results without needing to understand the presentation format of individual sources (eg, to remove duplicate documents). In Figure 3, a search is initiated from the user interface, which forwards a query (in XML) to the mediator. The mediator splits the query into several subqueries and sends these to the appropriate wrapper (via a capability manager if required). Finally, the wrapper translates the query into the native query language of the data source (eg, in HTML for Web data sources). Similarly, the result from the data source gets translated back into the system’s XML representation and sent back to the user interface.

##### Unified Query Language

UQL is used to represent queries obtained from users in a consistent internal way, and UQL statements identify query elements such as the external information sources to be searched and a set of search attributes used to delimit the search. For example, UQL expressions can store date range delimiters for a search. UQL also contains statements that indicate whether or not QC needs to process the query further. For example, we may wish to remove duplicate items obtained from different sources. In our current implementation, UQL is implemented using XML. To define the structure of the data within the XML document we use a data type definition (DTD), which allows various internal components of QC to validate the XML data received in the UQL query. The following example illustrates how a UQL query might look in XML.


<QUERY keyword = "iron AND deficiency"
  profile = "treatment"
  duplicateRemoval = "yes"
  sortBy = "rank"
  useLexicalVariants = "yes"
  timeout = "20"
  dateRangeBeginDay = "1"
  dateRangeBeginMonth = "1"
  dateRangeBeginYear = "1999" >
 <SOURCE name = "PubMed" />
 <SOURCE name = "Harrison’s online" />
 <SOURCE name = "Merck" />
 <SOURCE name = "MIMS" />
</QUERY>
                      

##### Unified Response Language

Similarly to the UQL, the unified response language (UReL) is used internally to guide display of information to users, also represented using XML. Each separate result, or “article,” from a source can be broken up into smaller chunks and given meta-data labels to represent the different sections of the data (eg, abstracts from journal articles). Since the majority of sources accessed by QC are journals, the data that are retrieved typically contain document elements such as Title, Author(s), Journal Name, Date of Publication, and the URL where the electronic version of the paper is accessed. Other sources, such as drug descriptions from pharmaceutical compendia, have sections such as Drug Name and Manufacturer. These different document elements, based upon the typical sources QC expects to find, are defined as specific fields in the UReL definition. The following example illustrates how a set of documents retrieved by QC might be represented in UReL.


<RESULT>
 <ARTICLE>
  <LINK>
   <HREF>
     http://www.ncbi.nlm.nih.gov:80
     /entrez/query.fcgi?cmd=Retrieve&db=PubMed
     &list_uids=12198020&dopt=Abstract
   </HREF>
   <LINKNAME>Abstract</LINKNAME>
  </LINK>
  <AUTHORLIST>Heath AL, 
    Skeaff CM, 
    Gibson RS.
  </AUTHORLIST>
  <TITLE>
    Dietary treatment of iron deficiency
  </TITLE>
  <DATE>
   <YEAR>2002</YEAR>
   <MONTH>9</MONTH>
  </DATE>
  <SOURCE>PubMed</SOURCE>
 </ARTICLE>
 <ARTICLE>
  <LINK>
   <HREF>
     http://mims.hcn.net.au
     /ifmx-nsapi/mims-data/?MIval=2MIMS_abbr_pi
     &product_code=288
     &product_name=Ferrum+H+Injection
   </HREF>
   <LINKNAME>More Information</LINKNAME>
  </LINK>
  <AUTHORLIST>
    Sigma Pharmaceuticals Pty Ltd.
  </AUTHORLIST>
  <TITLE>Ferrum H Injection</TITLE>
  <SOURCE>MIMS</SOURCE>
 </ARTICLE>
</RESULT>
                      

##### Wrappers

**Figure 4:**
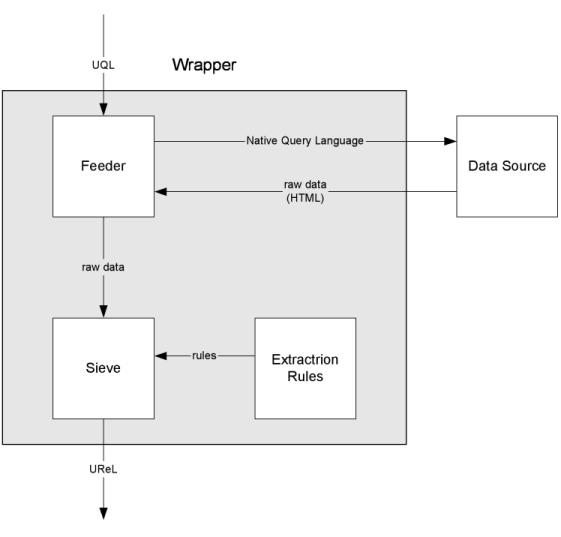
Wrapper components

For every information source known to QC, there is a specific wrapper that translates a UQL query into the native query language and format of the source. The wrapper also extracts the relevant information from the HTML result pages returned by the search engine and re-expresses it in UReL. Figure 4 shows the basic architecture of wrappers in our current system. Each wrapper has three main components: a feeder, extraction rules, and a sieve. The feeder converts the user query into the native query language of the data source. The data source responds to the query and returns HTML raw data. The feeder passes the raw data to the sieve, which converts it to UReL in XML format by using the extraction rules for the data source. The UReL is then sent back via other components to the user interface, which can interpret the XML and display the results.

##### Mediator

A key requirement of a multisource information retrieval system is the ability to perform concurrent searches on multiple sources with a single query [6,7]. The mediator addresses this requirement. The mediator first analyzes a query and determines how many sources are to be searched. It then creates a separate search job for each of these sources and forwards the search job to other system components. Additionally, the mediator collects individual results as they arrive and amalgamates them for the user into a single result. By introducing parallelism, the time to perform a search across a number of resources should be reduced to the duration of the slowest source. However, the potential drawback of parallel processing is the increased administration overhead of running multiple parallel processes within a system. As a rule of thumb, we would expect the benefits of parallel execution should increase with the number of sources queried, as response times for Web resources can be many seconds long, and computational execution of processes to manage parallel search are typically much less than one second.

Connection speed and latency of response time from sources are, for practical purposes, nondeterministic in an Internet environment, and a meta-search engine can therefore experience large fluctuations in responses from the same source under different circumstances. Latency is subject to network traffic conditions, making it impossible to guarantee that all resources that are queried at a particular time will respond predictably and equally. To counter this, the mediator has a time-out feature. If a response is not received within the time-out specified by a profile, the mediator will cancel a subsearch and forward all the results currently available from other sources to the user interface. This effectively guarantees a defined response time irrespective of the state of the individual data sources and provides some control over the speed/accuracy trade-off.

##### Capability Manager

Search capabilities vary considerably between the search engines that QC might wish to interrogate, and some sources will have limitations in their ability to process search queries. One approach to this problem is to try to raise all sources to as high a level of common performance as possible by emulating missing capabilities locally, usually by modifying the query and/or search result [13]. A trivial example is mimicking the ability to perform a Boolean search when a data source does not have this capability. To emulate a Boolean AND, a meta-search engine would perform two parallel individual searches on the source and then itself perform the Boolean operation on the two results.

In QC, a capability manager (CM) is responsible for mimicking a range of search capabilities and is located between the mediator and wrapper. The CM may modify a query and/or the result depending on the capabilities of the sources about to be queried. Capabilities of the CM within the QC system included the following:

- Date-CM: search within a date range
- Duplicate-CM: remove document duplicates
- Sort-CM: sort results by title, author, document rank, or date
- Lexical-CM: expand a search term with lexical variants of the term. A lexical variant is a synonym, pluralization, hyphenation, or other modification that changes the text but not its meaning. Lexical variants are particularly important in the medical domain [14] because many concepts can be expressed in Latin or English (eg, cardio vs heart). Moreover, there is a common confusion between terms in American English versus British English (eg, hemoglobin vs haemoglobin, epinephrine vs adrenaline).

QC uses a stacking mechanism to insert individual CMs into the processing of queries for wrappers and the processing of results from a source. A component called the search planner, containing simple rules, is responsible for stacking the CMs. This means that the sequence of CMs can be ordered to ensure the correct outcome of query or result translations. Theoretically, this corresponds to a composition of operations. A lexical variant CM, for example, has to replace the search terms in the query before the wrapper executes the search. The Date-CM, on the other hand, can only perform its job after the successful execution of the wrapper.

##### Search Filters

Expert searchers typically will use search strategies that are more likely to accurately locate information, based upon an understanding of the specific capabilities of an evidence source. There is an increasing interest in the writing of search filters which capture such strategies, usually focusing on the major evidence repositories like Medline [10,11]. Search filters are designed for typical clinical queries such as “diagnosis” or “prescribing,” and they are crafted to find evidence most likely to satisfy the query by first selectively searching resources identified to be of high quality and, second, by automatically adding specialist keywords to the general question posed by a user. Within QC, search filters are stored in the profiles function. For example, if a clinician selects the “diagnosis” filter and enters the search term “asthma,” QC can add in the additional terms when it queries Medline [10]:

sensitivity and specificity [MESH] OR sensitivity [WORD] OR diagnosis [SH] OR diagnostic use [SH] OR specificity [WORD]

These terms have been shown to significantly enhance the quality of Medline results, but they are unlikely to be known to a typical clinical user.

Unlike standard search filters, QC profiles are meta-search filters because they encode search filters for multiple different sources. Profiles thus encode expert search strategies that are most likely to answer a certain class of query, and they encode, among other things, the most appropriate content sources to search (Table 1). For a primary care physician, these search profiles might be for diagnosis, prescription, review, and treatment [4], but any set of profiles can be created within QC to meet the specific query types and search contexts of different users. In Table 1, the Treatment profile describes a set of nine separate source-specific search filters, which collectively describe the search strategy believed most likely to retrieve an accurate search result from each resource. The # symbol delimits keyword variables that are to be instantiated with user keywords. For example, #1# represents the keyword type “disease,” and QC’s mediator component will substitute the user-provided keywords for “disease” throughout the profile, prior to sending the query to the individual wrappers for the different sources. More than one search string can be created for an individual source (eg, TGL 1 and TGL 2) as a single strategy may not always retrieve all the relevant documents.

**Table 1:** Quick Clinical meta-search filters

**Source**	**Search String**
TGL1	(#1# AND #2# AND #3# AND #4#) AND+ ("treatment" OR "therapy" OR "therapeutic use")
TGL2	(#1# AND #3#) AND+ ("treatment" OR "therapy" OR "therapeutic use")
HealthInsite3	#1# AND #2# AND #3# AND #4#
HealthInsite4	#1# AND #3#
PubMed5	(#1# ATTR+ [Title] AND #3# ATTR+ [Title] AND #4# ATTR+ [Title] ATTR+ /ther) English 10 years Human
PubMed6	#1# ATTR+ [Title] AND (#3# ATTR+ [Title] OR #4# ATTR+ [Title]) ATTR+ /drug ATTR+ ther English 10 years Human
Merck7	((#1# AND #3# AND #4#) OR (#1# AND #3#)) AND+ ("treatment" OR "therapy")
Harrison’s8	Disconnected
Harrison’s9	Disconnected

##### System Platform

The system was constructed using Java, the Struts Web application framework, and a MySQL database and is deployed on a RedHat Linux platform. The user interface (JSP, servlet, and HTML pages) is deployed through an Apache Web server connected to a Tomcat servlet engine. The Apache-Tomcat platform incorporates load balancing and fail-over and is suitable for scalability and large-scale deployment.

#### Technical Evaluation

QC has undergone a series of clinical evaluations, which have been reported separately [4,15,16].

In total, 227 family physicians from across Australia participated in a trial of QC. Clinicians who had a computer with Internet access in their consulting rooms were recruited and asked to use QC for 4 weeks in routine care. Each participant was given a personal username and password to access the system. All clinicians completed an online pre-trial survey. QC was configured to search a set of eight sources, including remote sites such as PubMed, online journals such as *BMJ* and the *Medical Journal of Australia (MJA)*, and locally cached sources such as *The Merck Manual* and Therapeutic Guidelines Australia.

**Figure 5:**
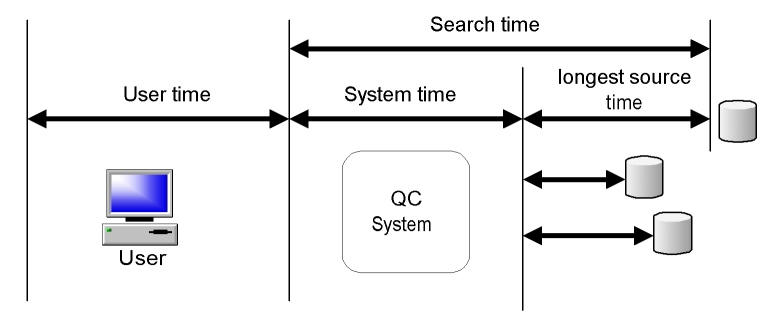
Search time metrics

For every search, the time from the request arriving at the system to the time when the results were sent back to the user was recorded (Figure 5; search time = system time + slowest source time). Note that there is a cap on search time when the time-out cuts in. Time-outs are search-profile dependent and were set at either 15 or 30 s. The time it took to conduct the search on the individual sources was also recorded. The time taken to send data between QC and the user’s computer (user time) is not incorporated in these measurements.

In the following section we report on the technical performance of the architecture and then reflect on its suitability for supporting evidence retrieval in clinical practice.

## Results

In the pre-trial questionnaire, 40% of the clinicians reported having a broadband (ADSL, cable, satellite) connection, while 43% used a 56k or 64k modem connection. The remaining 17% either did not know the type of connection used or had a slower connection. A total of 1662 searches were performed over the trial.

### Search Speed

Under local network conditions (LAN, 100MBit), the user time (from starting the search on a client computer to displaying the results) was approximately 1.5 s. However, since most users accessed the system through the Internet, latency was significantly longer and slowed down the overall search speed.

**Figure 6:**
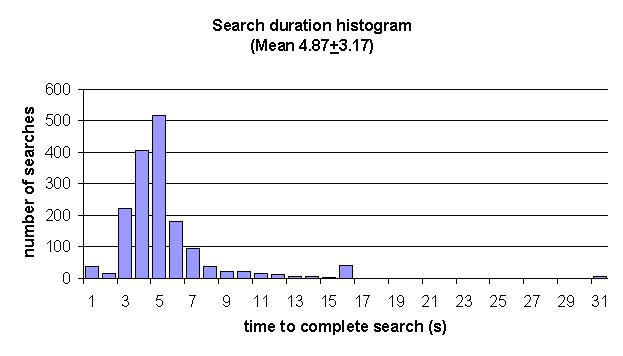
Distribution of search time for all 1662 searches

The average search time was 4.9 s, with a standard deviation of 3.2 s (N = 1662 searches). Figure 6 shows the distribution of all search times over the trial. There are four distinctive features in this chart. The first is a small peak at 1 s (ie, searches that took up to 1 s to complete). The second feature is a peak around the mean value. Third, there is a small peak at 15 s, and, fourth, there is a small peak at 30 s.

### System Time

**Figure 7:**
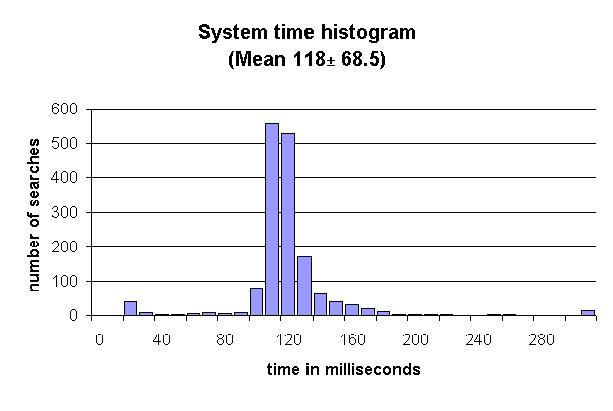
Distribution of the system time for 1614 searches

System time for a search was computed by subtracting the duration of the slowest source in every search from the search time (see Figure 5). From the system time histogram in Figure 7, it can be seen that for the majority of the searches the system takes between 100 ms and 130 ms (mean = 117.9 ms; SD = 68.4 ms; N = 1614 [48 searches had missing data, hence 1614 searches]).

### System Time Versus Number of Individual Sources Involved

Depending on the search profile selected, the system will query a certain number of information sources and combine the results. To illustrate the dependency between system time and the number of sources queried, Table 2 shows average system time versus the number of sources queried in a search. The number of sources queried is predefined by the search profile, and none of the search profiles tested queried five, six, or eight sources.

**Table 2:** System time vs number of sources queried

**Number of Sources Queried**	**N**	**Average System Time (ms)**
1	48	18.1
2	9	31.8
3	15	73.3
4	7	59.7
5	0	-
6	0	-
7	1373	122.2
8	0	-
9	162	122.6

### Speed and Reliability of Individual Data Sources

In addition to the performance measurements of the whole searches, the speed and reliability of the individual data sources was measured. Reliability was measured as the number of error cases (ie, queries that were not answered due to an error condition, such as a network error, an HTTP error, or queries that timed out). Reliability and speed figures are summarized in Table 3.

**Table 3:** Reliability and speed of data sources

**Source**	**Type**	**Number of Searches**	**Number of Errors**	**Error (%)**	**Mean Speed (s)**	**SD (s)**	**Min (s)**	**Max (s)**
Merck	Local	2144	0	0.0	0.06	0.11	0.01	2.89
TGL	Local	2193	0	0.0	0.05	0.12	0.01	2.85
BMJ	Remote	73	1	1.4	4.55	3.92	0.99	17.5
HealthInsite	Remote	2993	55	1.8	3.09	1.08	1.08	22.3
MedlinePlus	Remote	653	0	0.0	1.87	1.36	1.09	12.5
MIMS	Remote	650	3	0.5	0.98	1.14	0.28	8.30
MJA	Remote	58	1	1.7	0.25	0.31	0.10	1.73
PubMed	Remote	3288	39	1.2	3.76	1.69	1.87	15.0
Total		12052	99					
Mean				0.8	1.83	0.63*	0.68	10.4

^*^ standard error of the mean

**Figure 8:**
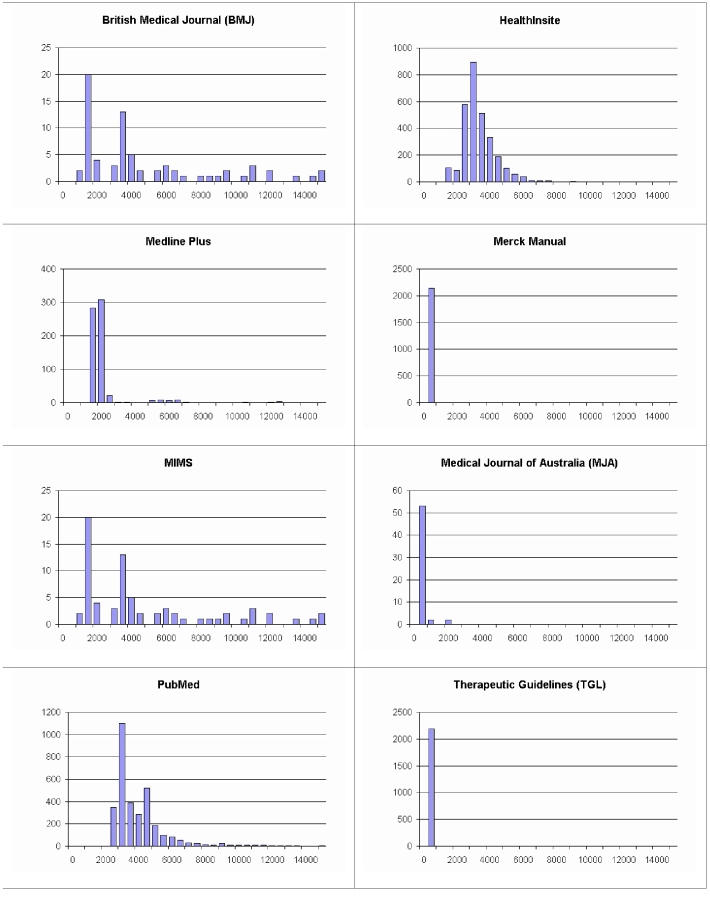
Histogram of search times for each of the eight data sources (x-axis is time taken for a search, in ms; y-axis is number of searches)

The most reliable sources were the locally indexed sources Merck (*The Merck Manual*) and TGL (Therapeutic Guidelines Australia), both which did not have any error cases. On the other end of the scale are HealthInsite (a national consumer site for health information) and *MJA*. The slowest source in the trial was *BMJ*, with an average of 4.55 s to process a query (SD = 3.92 s; N = 73). This was followed by PubMed, which returned results at an average of 3.76 s (SD = 1.69 s; N = 3288). The two locally indexed sources (Merck and TGL) returned search results within an average of 0.061 s and 0.047 s, respectively. However, the two local sources do have a relatively large standard deviation. Figure 8 shows the distribution of query times to the eight individual data sources.

## Discussion

### System Time

From the results of the system time versus source time, we can observe that system-processing time is only a fraction of the total search time. However, there are exceptions, namely when local data sources are used exclusively. From a user’s perspective this still would not be an issue as the overall user time is greater by at least a factor of ten. It could, however, become a problem in a situation where many searches are dependant on the result of a previous search and have to be executed in series. System time has thus been kept relatively short, removing the initial reservation that too much parallelism could slow down the system excessively. From Table 3 it can be seen that the system time generally increases in line with the number of sources queried (with the exception of four sources queried). However, the order of this increase does not appear to be squared or even exponential, but rather linear.

### Search Times

The four distinct features in the histogram of search times described in Figure 6 are due to the nature of the data sources and the value of the time-outs. The first small peak at 1 s is from search profiles that use exclusively local data sources. The second feature is a peak around the mean value and is caused by the six Internet resources. The small peak at 15 s is due to the large number of search profiles that have this value as a time-out. And finally, the tiny peak at 30 s is where the remaining searches time out.

It was to be expected that local sources would be more reliable and have a shorter latency in response time. This is due to the controlled environment, compared to the uncontrolled Internet environment of the external sources. It is interesting to note the difference between the six external data sources. While some sources are very popular (eg, PubMed) and therefore are expected to be busy, others might lack the resources to keep up with demand. The time-out value of individual data sources is a trade-off between speed and quality of results and is determined by the intended usage of the system. However, under certain circumstances there are optimizations that can be carried out without affecting quality of results. For example, the search duration histogram for HealthInsite (Figure 8; top right) reveals that if a search has not completed within 10 s it is highly unlikely it will complete within 15 s. Therefore, a time-out value of 15 s can safely be reduced to 10 s without significantly compromising search quality.

### Future Work

The current QC architecture has demonstrated in trials that it meets the technical design goals set for it, and it provides good evidence that our general approach to federated searching is sustainable and maintainable. We intend to pursue research and development in areas of current interest to meta-search engines, information retrieval systems, and artificial intelligence. These include automatic wrapper generation [17,18] so that new data sources can be easily integrated into QC. Using this approach, a component could automatically generate a wrapper from knowledge of the data source query inputs and results. Another area of continued research will be automated data source consistency checking. Data sources often change in their formats, and this needs to be monitored with either automated or human intervention in order to modify wrappers accordingly. A third area will be intelligent search agents [19]. We envisage incorporating an intelligent agent that will guide users through the search process, using domain knowledge to help frame clinical questions and choose search parameters. This agent could learn to work with its user. An area of continued development will be semantic understanding of result sets. We would like QC to combine search results into a meaningful coherent story that presents a concise, relevant, and digestible response to the user [20]. These approaches, coupled with user support, will allow us to develop and improve the system with a view to it becoming an integral part of a clinician’s daily practice. Even without these enhancements, we have demonstrated that the QC framework is a functional and useful approach for the delivery of online, just-in-time clinical evidence.

## Abbreviations

CM: capability manager
QC: Quick Clinical
UQL: unified query language
UReL: unified response language
